# Effectiveness of a Cognitive Behavioral Therapy for Dysfunctional Eating among Patients Admitted for Bariatric Surgery: A Randomized Controlled Trial

**DOI:** 10.1155/2014/127936

**Published:** 2014-07-21

**Authors:** Hege Gade, Jøran Hjelmesæth, Jan H. Rosenvinge, Oddgeir Friborg

**Affiliations:** ^1^Morbid Obesity Center, Vestfold Hospital Trust, P.O. Box 3168, 3103 Tønsberg, Norway; ^2^Department of Psychology, University of Tromsø, P.O. Box 6050 Langnes, 9037 Tromsø, Norway

## Abstract

*Objective.* To examine whether cognitive behavioral therapy (CBT) alleviates dysfunctional eating (DE) patterns and symptoms of anxiety and depression in morbidly obese patients planned for bariatric surgery. *Design and Methods*. A total of 98 (68 females) patients with a mean (SD) age of 43 (10) years and BMI 43.5 (4.9) kg/m^2^ were randomly assigned to a CBT-group or a control group receiving usual care (i.e., nutritional support and education). The CBT-group received ten weekly intervention sessions. DE, anxiety, and depression were assessed by the TFEQ R-21 and HADS, respectively. *Results.* Compared with controls, the CBT-patients showed significantly less DE, affective symptoms, and a larger weight loss at follow-up. The effect sizes were large (DE-cognitive restraint, *g* = −.92, *P* ≤ .001; DE-uncontrolled eating, *g* = −.90, *P* ≤ .001), moderate (HADS-depression, *g* = −.73, *P* ≤ .001; DE-emotional eating, *g* = −.67, *P* ≤ .001; HADS-anxiety, *g* = −.62, *P* = .003), and low (BMI, *g* = −.24, *P* = .004). *Conclusion.* This study supports the use of CBT in helping patients preparing for bariatric surgery to reduce DE and to improve mental health. This clinical trial is registered with NCT01403558.

## 1. Introduction

Bariatric surgery may result in significant weight loss, however with large individual differences [1, 2]. In patients eligible for bariatric surgery (BS), dysfunctional eating (DE) has been found among 10–25% of obese patients considered for or completing bariatric surgery [3, 4], and DE has been reported both prior [3–6] to and after BS [7–10]. DE can be operationalized as exerting rigid control, or loss of control over eating, or eating for emotional reasons rather than hunger or appetite. DE, in particular emotionally regulated eating, may be negatively reinforced if used to alleviate negative mood or feelings of stress [11].

DE is associated with overconsumption of energy dense food [12–15], which may impair sustained weight loss postsurgically [7, 11, 16–18]. Conversely, psychological treatments which target DE may increase the possibility of sustained weight loss following BS.

In addition to DE, patients with morbid obesity may suffer from symptoms of anxiety and depression. The prevalence of any mood disorder is about 16% and 24%, respectively [19]. Theoretically, improving affective symptoms might improve control over eating as there are fewer negative affects that one needs food to regulate. Moreover, alleviations in depression may facilitate experiences of self-efficacy and hence the motivation to implement the necessary behavioral changes in terms of adhering to dietary recommendations [20]. Both disorders may be effectively treated by cognitive behavioral therapy (CBT) [21].

To our knowledge, no previous controlled studies have tested the efficacy of a CBT-intervention aimed at reducing DE in obese patients selected to BS. However, several sources of knowledge indicate that such an intervention could be feasible. The convincing body of knowledge from controlled trials has established CBT as the treatment of choice for the spectrum of eating disorders according to diagnoses and clinical severity [22] including binge eating disorder (BED) [21]. DE may be considered as a milder variant of BED. Hence, a treatment working for the severe variant should logically also work for the milder one. Other sources of knowledge come from a case study of a patient admitted to BS [23] as well as from uncontrolled pre-post studies of larger series of patients, indicating that CBT might be an appropriate approach [21, 24].

Using a randomized controlled design, the purpose of this study was to examine the efficacy of a CBT-intervention in improving DE as well as affective symptoms. We hypothesized that the intervention would be superior to usual care, particularly with respect to reducing emotional and uncontrolled eating and increasing cognitive restraint of eating.

## 2. Methods

### 2.1. Participants

A total of 102 eligible (69 females and 33 males) consecutive morbidly obese patients admitted for bariatric surgery agreed to participate. All patients participated based on informed consent.

### 2.2. Study Design

This randomized controlled trial (http://clinicaltrials.gov/ct2/show/NCT01403558) used a mixed design: one between-group factor (intervention versus usual care) and one within-group factor (pre- and postmeasures). The time-interval between pre- and postmeasurements was 10 weeks.

### 2.3. Randomization

A block randomization procedure (http://www.randomizer.org) was employed (with blocks of 4) to ensure balance between the groups. Two research assistants at the treatment center, with no affiliation to the study, had access to the key to the randomization file. After having read and signed the informed consent letter and completed the baseline measurements, the patients as well as the first author were informed about the allocated treatment arm. The allocation ratio was 1 : 1.

### 2.4. Procedures before Surgery (All Patients)

During the four months prior to surgery, patients in both treatment arms were offered up to three consultations from either a medical doctor, a dietician, a nurse, or a physiotherapist. These consultations were voluntary and were based on the patients' individual needs. Here the patients received educational materials concerning nutritional recommendations, detailed information about the mandatory low calorie diet the last three weeks before surgery, and guidance about recommended physical activity level and intensity.

### 2.5. Intervention Group

The patients in the intervention group received ten sessions based on theoretical principles from CBT, that is, learning to recognize triggers of DE, identifying associated cognitions and emotions, initiating plans for change, and use of home-work task in between the sessions. Sessions 1-2 included strategies to enhance intrinsic motivation and addressed resistance to change [25]. Sessions 2–11 were based on CBT-principles.  Table 1 provides an overview of the contents of all sessions. Five sessions were carried out at the treatment center, and the remaining six as scheduled telephone calls.

### 2.6. Measurements and Outcomes

Demographic and clinical variables comprised age, gender, educational level, employment, and BMI.

### 2.7. Dysfunctional Eating (DE)

The primary outcome measures were changes in DE as measured by the Three-Factor Eating Questionnaire (TFEQ R-21) which has been validated for use in obese individuals [26, 27]. It consists of 21 items comprising three subscales: “emotional eating” (EE; 6 items; Cronbach's *α* = .92), “uncontrolled eating” (UE; 9 items; *α* = .73), and “cognitive restraint of eating” (CR; 6 items, *α* = .84). According to the manual, the three subscales were transformed to a 0–100 scale to become comparable [26]. Higher scores indicated more severe dysfunction. The reliability of the subscales in the present study was comparable to previous reports [26].

### 2.8. Affective Symptoms

Secondary outcome measures were symptoms of anxiety and depression, measured by the Hospital Anxiety and Depression Scale (HADS) [28]. HADS is a self-report measure of nonvegetative affective symptoms [28, 29] where seven items assess depression (HADS-D) and seven items measure anxiety (HADS-A), respectively. Items are scored 0–3 yielding a range of 0–21 within each subscale. A cut-off ≥8 is used in Norway to indicate a clinically probable impairment due to depression or anxiety [30]. Cronbach's alphas for HADS-A and HADS-D were .84 and .78, respectively.

The procedures were initiated after the study had been approved by the Regional Committee for Medical and Health Research Ethics (2010/2071a).

### 2.9. Sample Size 

Based on clinical experience, reductions in the emotional and uncontrolled eating scores of 15% or more were considered to be clinically meaningful. A conservative estimate was that no patients in the control group, and at least 30% in the intervention group, would achieve this treatment goal. Given this difference in treatment effect, a 90% statistical power, a significance level of 5%, and a dropout rate of 40%, a minimum sample size of 80 patients was required. To allow for a 20% withdrawal rate, we included 102 patients in the current study. The statistical power was excellent for all analyses (>.99).

### 2.10. Statistical Methods

Data were analyzed by the Statistical Package of the Social Science (SPSS) for Windows, version 17 (SPSS, Chicago, IL, USA).

The intervention effects were examined by analysis of covariance (ANCOVA), comparing the two posttest group mean scores adjusted for baseline scores. Effect sizes were reported as Hedges' *g* indicating the differences between the groups in number of standard deviations. Effect sizes of 0.20, 0.50, and 0.8 were regarded as small, moderate, and large [31].

## 3. Results

### 3.1. Recruitment and Participant Flow

**Figure 1:**
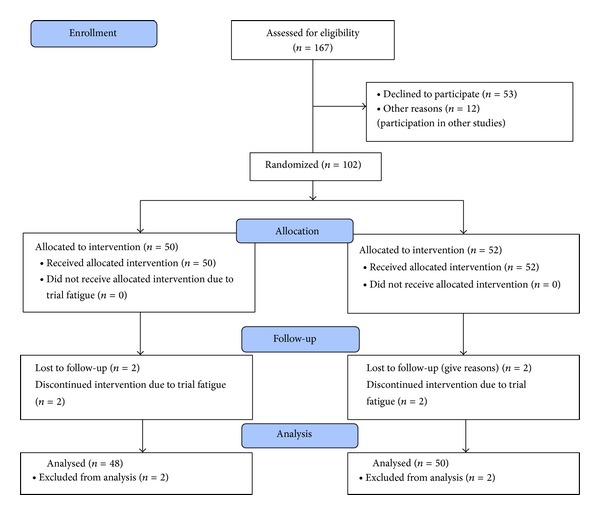
Participant flow (morbidly obese patients admitted for bariatric surgery).

Hundred and two patients agreed to participate; four patients were lost to follow-up, leaving data from 98 patients for analysis (Figure 1). A completers-only analysis was conducted at follow-up as attrition was minor.

### 3.2. Baseline Data

Clinical baseline data are presented in Table 2 showing that most participants (82%) had finished upper secondary school (≥12th grade), 54% were employed, and 40% received disability pension or a temporary pension while assessing work ability.

The prevalences of clinically relevant symptoms of anxiety and depression (HADS ≥ 8) were 41% and 25%, respectively.

### 3.3. Effect of the Intervention

The patients in the CBT-group had significant improvements in DE, anxiety, and depression compared with the control group patients. A significant reduction in BMI was also observed.

**Figure 2:**
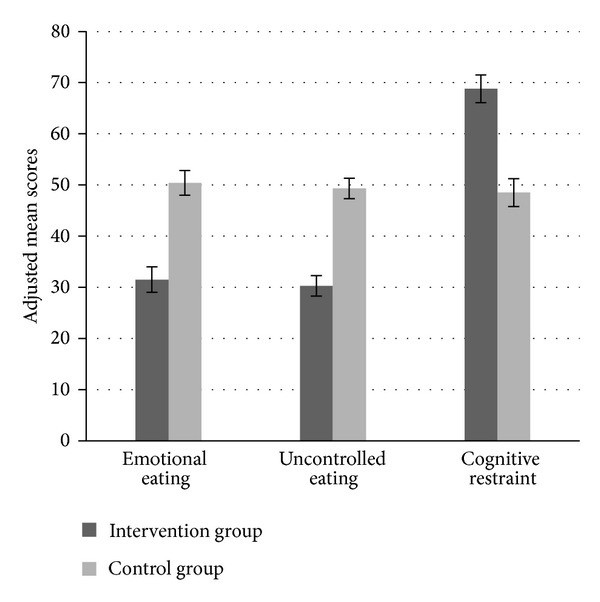
Postintervention (10-week) scores for eating behaviors by treatment arm. Data expressed as adjusted mean scores. Error bars expressed as standard errors of the mean. The Three-Factor Eating Questionnaire (TFEQ R-21) was used to measure the three domains of eating behaviors.

**Figure 3:**
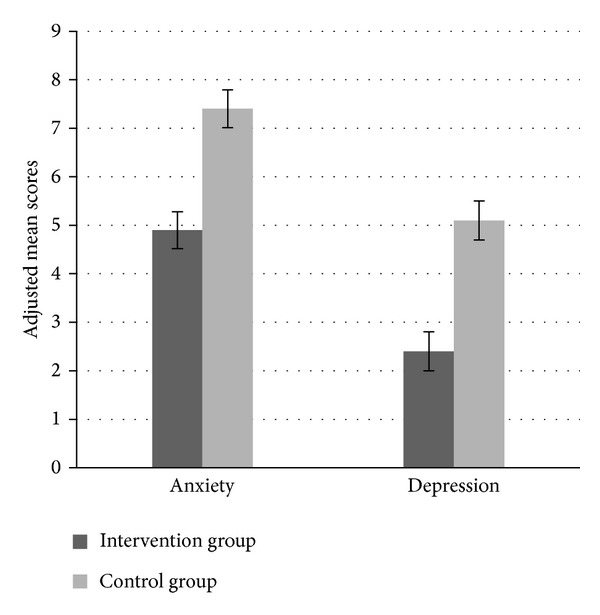
Postintervention (10-week) scores for anxiety and depression by treatment arm. Data expressed as adjusted mean scores. Error bars expressed as standard errors of the mean. The Hospital Anxiety and Depression Scale (HADS) was used to measure anxiety and depression.

The intervention effects are presented in Figures 2 and 3 showing postinterventional scores for eating behaviors and affective symptoms by treatment. The between-group effect sizes for the improvements varied from high (uncontrolled eating (*g* = − .90, *P* ≤ .001), cognitive restraint (*g* = .92, *P* ≤ .001)) to moderate (emotional eating (*g* = − .67, *P* ≤ .001), anxiety (*g* = − .62, *P* ≤ .001), depression (*g* = − .73, *P* =  ≤.001)) and low (BMI (*g* = − .24, *P* = .004)).

Adjusted between-group differences at follow-up for EE, UE, and CR were −19 (95% CI, −26 to −12), −19 (95% CI, −25 to −14), and 20 (95% CI, −28 to −13), respectively, all *P* ≤ .001. For anxiety and depression the adjusted between-group differences were −2.5 (95% CI, −3.5 to −1.4) and −2.8 (95% CI, −3.9 to −1.6), respectively, both *P* ≤ .001. Concerning BMI and body weight, the adjusted between-group differences were −1.1 kg/m^2^ (95% CI, −1.8 to −.35, *P* = .004) and −3 kg (95% CI, −5.1 to −.84, *P* = .004).

## 4. Discussion

This study contributes to the literature as being the first randomized controlled trial of a CBT-intervention to treat dysfunctional eating behaviors among severely obese patients scheduled for bariatric surgery. It demonstrated that patients in the CBT-group showed a strong reduction in DE and a moderate alleviation of anxiety and depression following the 10-week intervention compared to the control group. In addition, the CBT-group lost about 3 kg body weight.

To our knowledge, no previous controlled study has assessed a CBT program in the treatment of DE. Nevertheless, DE is closely linked to BED both cognitively and behaviorally in terms of eating patterns and the use of food to regulate negative mood. Although BED was not assessed in the present study, a comparison with previous BED-studies may be warranted. Hence, previous BED-studies [21, 24] support our findings in the sense that a CBT-intervention effectively reduced binge eating symptoms and associated cognitions, as well as increasing postoperative weight loss. On the other hand, comparisons across studies may be difficult due to incomplete treatment descriptions [21] and divergent study designs [24].

As DE often includes overeating in relation to negative mood states or a tendency to lose control over eating, it includes cognitive, emotional, and behavioral elements. It is plausible that a targeted CBT-program could have beneficially affected all these elements. As repeated measures following each session were not taken, it was impossible to discern which of the specific parts of the intervention produced the improvements. In addition, a nonspecific, independent effect of the therapist and the therapeutic alliance cannot be excluded.

The reduction of affective symptoms might be considered an adjuvant effect of the CBT-intervention. Although the intervention did not address symptoms of poor mental health specifically, it addressed how to detect and improve the tolerance of negative emotions triggering DE behaviors and associated dysfunctional cognitions. Moreover, as the intervention included home-sessions with practical tasks that were possible to accomplish for all patients, feelings of coping and mastery might partly explain the apparent antidepressive and anxiety reducing effect.

Strengths of the study are the randomized controlled treatment design and the low attrition rate. In addition, the recruitment of consecutive treatment seeking white morbidly obese patients preparing for bariatric surgery in a large tertiary care center suggests that our results may be generalizable to similar populations.

As the first trial addresses DE and the effect of CBT before bariatric surgery, our results need replication trials. Indeed, such replications will need to take this pioneer study's limitations into account. Notably, one needs to sort out common versus specific effects by including more than one therapist as well as possible treatment component effects. In addition, further studies should develop a control treatment condition in more detail and with a number of sessions equal to the CBT-condition. Furthermore, future studies should also collect additional data such as binge eating symptoms, which was not done in the current study.

This study shows the success of a 10-week CBT-intervention program in improving DE behaviors and affective symptoms in morbidly obese patients admitted for bariatric surgery. Future research should investigate whether these proximal effects are sustained and whether presurgical improvement in DE behaviors and affective symptoms do provide an additive benefit to bariatric surgery in terms of a stabilization of weight loss.

## Figures and Tables

**Table 1:** Overview of the 10-week CBT-intervention.

Sessions	Session content
Session 1 *(at the center) both groups *	(i) Establishing rapport with the patient in order to facilitate a good therapeutic working alliance. (ii) Providing information about the interventions to all patients. (iii) Conducting the baseline measurements and performing the randomization and informing the patients about their allocated group.

Session 2 *(at the center) *	(i) Introduction to the underlying principles of the therapy (working transparently, collaboratively, being time-limited, and using a manual). (ii) Informing the patient about CBT and the treatment plans in the study. (iii) Psychoeducation focusing on the relationships between eating behaviors, cognitive and behavioral patterns, affect-regulation, and obesity, thus introducing the patients for the CBT model. (iv) Introducing and explaining home-work sheets for sessions 3 and 4.

Sessions 3 + 4 *(by telephone calls) *	(i) Reviewing the patient's home-work sheets. (ii) Recognizing and addressing dysfunctional eating behaviors. (iii) Working with the patient's behavioral eating patterns (what triggers eating), and the associated cognitions and emotions. (iv) Providing the patients' means to assess their own perception about recognizing improvement in dysfunctional cognitions and eating behaviors.

Session 5 *(at the center) *	(i) Coping with situational “triggers” that may lead to dysfunctional cognitive and eating behavioral patterns. (ii) Working with the patient's cognitive and behavioral eating patterns (“triggers,” cognition, emotion, and eating behavior). (iii) Introducing and explaining home-work sheets for sessions 6 & 7.

Session 6 & 7 (*by telephone calls) *	(i) Reviewing the patient's home-work sheets. (ii) Continuing the intervention techniques. (iii) Reinforcing positive changes in eating behaviors.

Session 8 *(at the center) *	(i) Continuation or refining intervention techniques (as session 5) by guiding the patient in avoiding situational “triggers” and making a plan for practicing new eating behaviors. (ii) Introducing and explaining home-work sheets for sessions 9 & 10.

Session 9 & 10 (*by telephone calls) *	(i) Reviewing the patient's home-work sheets. (ii) Continuation or refining intervention techniques.

Session 11 *(at the center) *	(i) Relapse prevention. (ii) Ending of treatment and helping the patient to maintain positive changes.

**Table 2:** Baseline demographics, eating behaviors, anxiety, and depression among 102 patients admitted for bariatric surgery by treatment arm.

	Total (*n* = 102)	Intervention (*n* = 50)	Controls (*n* = 52)
BMI (kg/m^2^)	43.5 (4.9)	43.6 (5.1)	43.5 (4.7)
Weight (kg)	128.0 (19.1)	129.1 (18.0)	126.9 (20.1)
Gender			
Female	69	31	38
Male	33	19	14
Age (years)	42.6 (9.8)	44.1 (9.8)	41.2 (9.6)
Educational level			
<12th grade	84 (82.4)	41 (82.0)	43 (82.7)
High school/college degree	18 (17.6)	9 (18.0)	9 (17.3)
Employment			
Employed	55 (53.9)	26 (52.0)	29 (55.8)
Unemployed	6 (5.9)	3 (6.0)	3 (5.8)
Temporary pension	21 (20.6)	11 (22.0)	10 (19.2)
Disabled	20 (19.6)	10 (20.0)	10 (19.2)
Eating behaviors			
Emotional eating	52.4 (26.0)	53.4 (27.1)	51.4 (25.0)
Uncontrolled eating	49.0 (18.8)	50.5 (17.7)	47.4 (20.0)
Cognitive restraint	44.1 (20.5)	42.7 (19.7)	45.5 (21.2)
Affective symptoms			
Anxiety	6.7 (3.9)	7.0 (4.2)	6.5 (3.7)
Depression	5.1 (3.4)	5.5 (3.7)	4.7 (3.0)

*Number (%) or mean (SD). *The Three-Factor Eating Questionnaire (TFEQ R-21) was used to measure the three domains of eating behaviors, and the Hospital Anxiety and Depression Scale (HADS) was used to measure anxiety and depression.
